# Lower button‐cortex distance and lower revision rates with adjustable‐loop compared to fixed‐loop cortical suspension devices for anterior cruciate ligament reconstruction

**DOI:** 10.1002/jeo2.70212

**Published:** 2025-03-22

**Authors:** Ron Gilat, Amit Gilad, Ilan Y. Mitchnik, Yoav Comay, Assaf Moriah, Gabriel Agar, Yiftah Beer, Dror Lindner

**Affiliations:** ^1^ Department of Orthopaedic Surgery Maimonides Medical Center New York New York USA; ^2^ Faculty of Medical and Health Sciences Tel Aviv University Tel Aviv Israel; ^3^ Department of Orthopaedic Surgery Shamir Assaf Harofeh Medical Center Be'er Ya'akov Israel

**Keywords:** ACL reconstruction, adjustable‐loop, button, cortical suspension device, fixed‐loop

## Abstract

**Purpose:**

To compare button position following femoral fixation of an anterior cruciate ligament (ACL) graft using fixed‐loop cortical suspension device vs. an adjustable‐loop device. Subsequently, to assess the association of button position‐related factors and revision ACL reconstruction.

**Methods:**

This was a retrospective cohort study of consecutive patients undergoing ACL reconstruction using fixed‐loop (Endobutton CL) and adjustable‐loop cortical suspension device (Ultrabutton) for femoral fixation in a single institution between 2009 and 2022. Demographic and operative characteristics were recorded. To assess soft tissue interposition the distance between the button and the lateral femoral condyle (LFC) was measured on X‐rays made on the first post‐operative day. Other measurements included button angle, relative position (anterior/middle/posterior), and button migration (assessed using most recent X‐rays).

**Results:**

Overall, 244 patients were included in the study. 59% of patients in the fixed‐loop group and 41% in the adjustable‐loop group. Hamstrings autograft was utilised most commonly (91%), while the rest of the procedures included allografts. A significantly shorter button distance from the LFC was noted in the post‐operative Antero‐posterior (AP) X‐ray of the adjustable‐loop button, 0.44 ± 0.52 mm versus 0.72 ± 0.84 mm, respectively (*p* = 0.002). Revision rates were significantly lower in the adjustable‐loop group (4%) versus the fixed‐loop group (12%, *p* = 0.035). No statistically significant direct association was found between button distance from the LFC and revision ACL reconstruction.

**Conclusions:**

Adjustable‐loop cortical suspension devices for femoral fixation of an ACL reconstruction were associated with lower revision rates and a lower button‐LFC distance when compared to fixed‐loop devices.

**Level of Evidence:**

Level III, retrospective comparative cohort study.

**Abbreviations**
ACL: anterior cruciate ligament
ACLR: anterior cruciate ligament reconstruction
AP: antero‐posterior
ICC: inter‐observer correlation
ICD: International Classification of Diseases
LAT: lateral
LFC: lateral femoral condyle
MFX: microfracture
MMx: medial meniscectomy
OR: odds ratios
PCL: posterior cruciate ligament

## INTRODUCTION

Anterior cruciate ligament (ACL) tear is one of the most common and devastating knee injuries in patients engaged in sports activities [5]. ACL reconstruction is a common procedure performed to restore stability and function to the knee joint following ACL injury, and by some believed to prevent early knee osteoarthritis [1, 2, 12]. The femoral fixation and tunnel location of ACL grafts is an important aspect of the procedure, as it determines the initial position and stability of the graft [8], therefore it may also influence success of treatment and failure rates. There are several options for femoral fixation of an ACL graft during ACL reconstruction, including suspensory extracortical buttons, interference screws, and transfemoral pins [11]. Two common types of suspensory extracortical buttons are the fixed loop‐cortical button and the adjustable‐loop cortical button [3].

Soft‐tissue interposition between the aforementioned types of buttons and the femoral condyle can potentially lead to decreased graft tension and function resulting in instability, an increased risk of graft rupture, and lower patient‐reported outcomes [10]. 25% of fixed‐cortical devices were deployed with soft‐tissue interposition seen on X‐rays one day after surgery. This phenomenon often subsequently results in button migration [6]. The use of direct visualisation of the button to confirm the correct deployment of the button, has been suggested [6], and using intraoperative fluoroscopy to confirm button position was also studied [9]. O'Brien et al. [9] found 15.7% had interposed soft tissue and 9.8% had an improperly flipped button. They concluded that while deployment errors are common, their correction is not technically demanding. However, Ozbek et al. [10] have evaluated 249 patients who underwent single‐bundle ACL reconstruction with hamstrings autografts. They found differences between patients in soft tissue interposition and suspensory cortical button migration did not significantly affect postoperative clinical or functional outcomes or graft ligamentization [10].

The literature regarding the incidence of incorrect deployment and the position of these devices following ACL reconstruction is still limited. The purpose of this study is to compare button position following femoral fixation of an ACL graft using fixed‐loop buttons (Endobutton CL, Smith and Nephew, Andover, MA, USA) versus adjustable‐loop buttons (Ultrabutton, Smith and Nephew). Subsequently, we aimed to assess the association of button‐related factors and revision ACL reconstruction. We hypothesised that button position elements and revision rates would be similar between the two groups.

## METHODS

### Study design, setting and study population

This was a retrospective comparative cohort study aimed to investigate the outcomes of ACL reconstruction using fixed‐loop button and adjustable loop button femoral fixation techniques. The study was conducted at a single institution between the years 2009 and 2022. An institutional review board (ASF‐0021‐23) approved the study prior to its commencement.

### Data collection, variables and measures

Data was obtained by performing a search for an ICD9 Diagnosis (International Classification of Diseases) 81.45 of ACL reconstruction procedure performed in a single institution within the determined period. Inclusion criteria included primary ACL reconstruction using hamstrings autograft or soft tissue allograft and age at time of surgery >15 years old. Exclusion criteria were as followed: revision ACL reconstruction, other ACL grafts, missing data, missing or poor‐quality postoperative X‐rays, ACL Re‐insertion, multi‐ligament reconstruction, and double‐bundle ACL reconstruction techniques. Each patient's operative report and postoperative X‐rays were assessed meticulously to confirm it was suitable and met the inclusion and exclusion criteria. Demographic and operative data were retrieved from electronic patient records for all consecutive patients who underwent ACL reconstruction with either fixed‐ or adjustable‐loop fixation. Variables included age, gender, laterality, time to surgical intervention, ACL rupture type (complete or partial), graft type (autograft or allograft) and any additional procedures performed during knee arthroscopies.

### ACL reconstruction surgical technique

**Figure 1:**
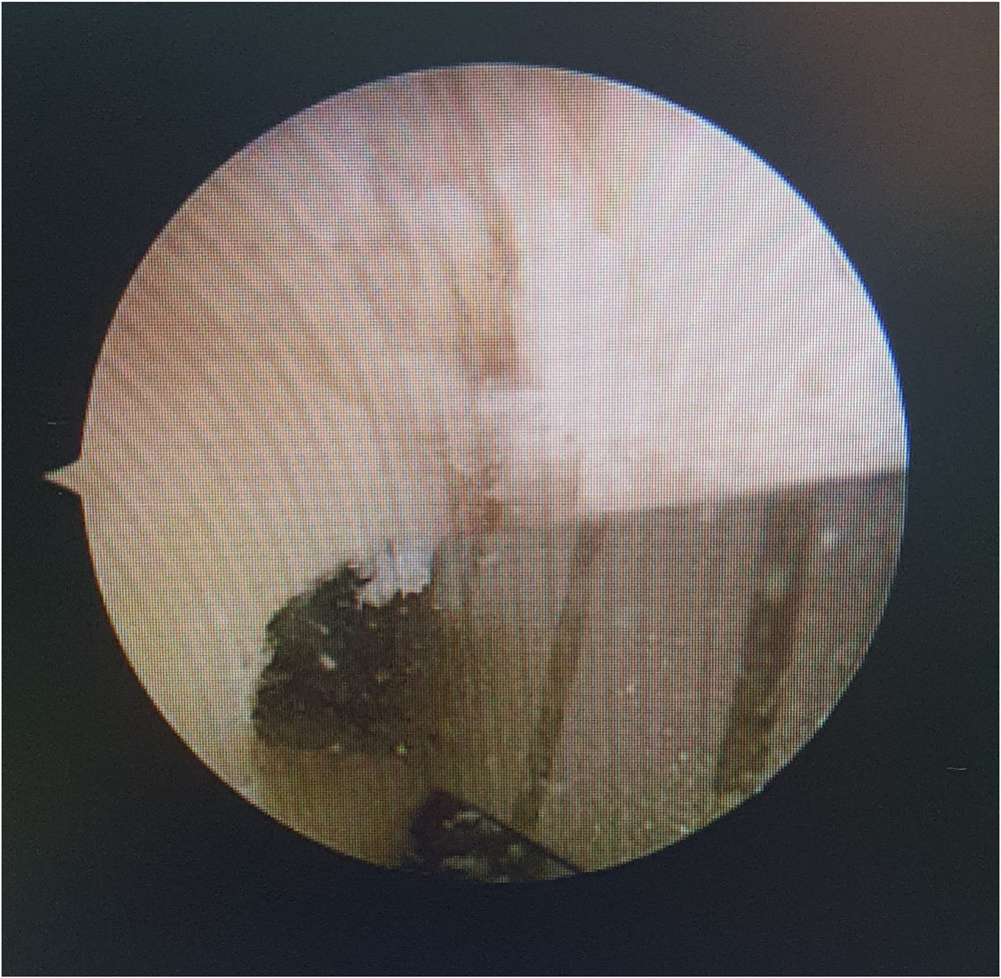
Intra‐operative measurement of femoral tunnel length after 4.5 mm drilling.

**Figure 2:**
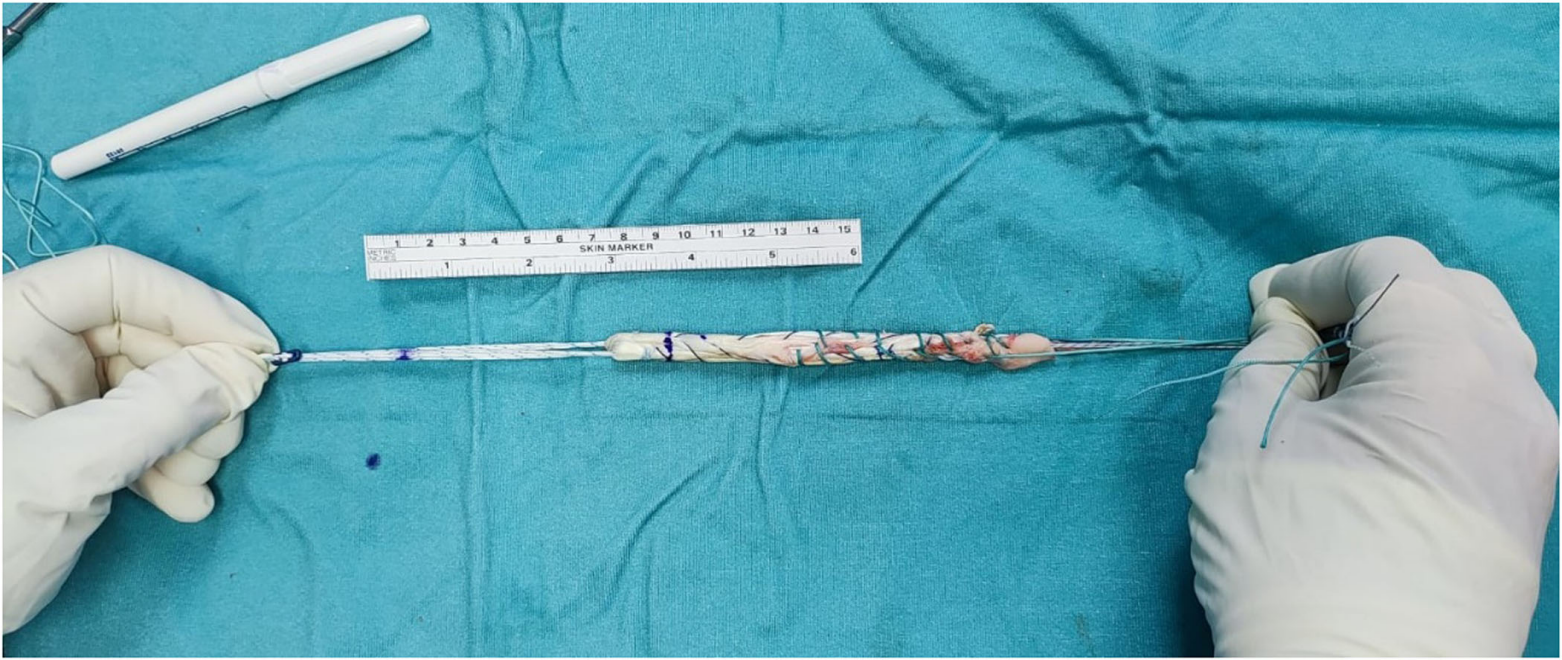
Graft marking method using an adjustable‐loop button. The marking on the white sutures reflects the 4.5 mm tunnel length, the marking on the graft itself reflects the depth of the fully reamed tunnel (the part of the graft which would be interposed in the bone tunnel).

**Figure 3:**
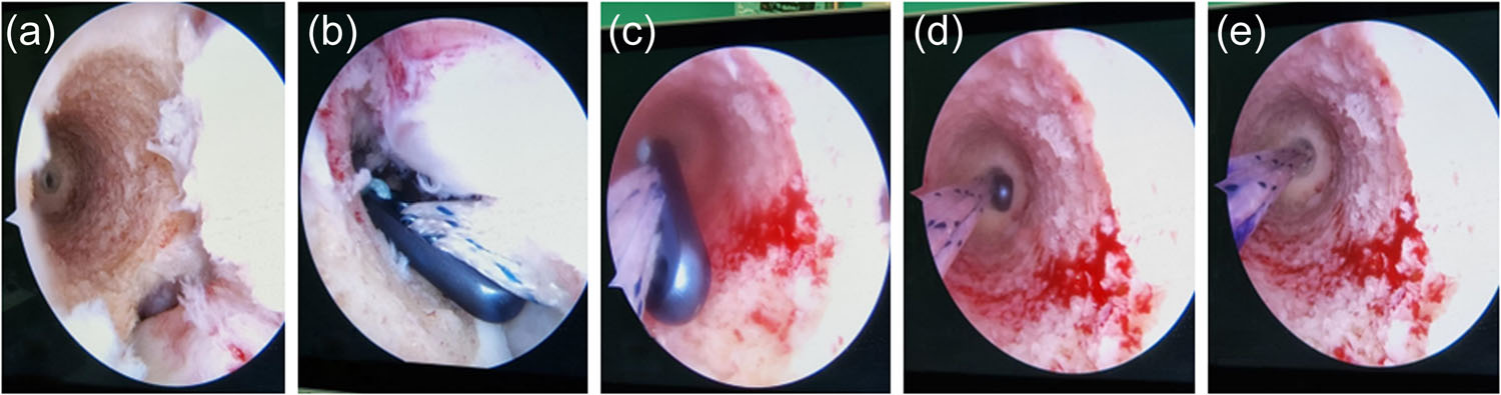
(a–e) Adjustable‐loop button (Ultrabutton) deployment technique. Femoral tunnel as viewed from the antero‐medial (AM) portal. (a) “Eye in the sky” view, verifying intact walls, clearance of the tunnel from debris, and patency of the 4.5 mm center hole at the bottom of the socket. (b) Passage of a button into the tunnel. (c) The button is pulled using the green leading suture, while the white suture is slightly tensioned just enough to accompany the green suture, but not tension the adjustable loop. (d) The button is viewed passing vertically into the 4.5 mm tunnel. (e) The button is flipped as it reaches the lateral cortex of the lateral femoral condyle (LFC). This is done slowly, by feel and by viewing of the purple mark on the white suture loop as it reaches the entry of the socket (the mark represents the length of the 4.5 mm tunnel as measured beforehand arthroscopically). This technique allows both direct visualization of the button until its deployment and also minimises the risk for entrapment of soft tissue between the button and lateral cortex of the LFC.

Our standard surgical technique for ACL reconstruction (ACLR) includes a standard arthroscopic knee evaluation of the patellofemoral joint, medial and lateral compartments including cartilage and meniscus, ACL and posterior cruciate ligament (PCL) integrity. Next, if using Hamstring autograft, the graft is harvested including Gracilis and Semitendinosus tendons through a standard anteromedial approach. While the graft is being prepared, meniscus and cartilage pathology are treated in a standard manner, and tunnels for the graft are made, the anatomical insertion of the native ACL in the medial part of the lateral femoral condyle (LFC) is cleared using a shaver and radiofrequency devices, first, a beath pin is drilled through the lateral cortex using an offset guide from the anteromedial portal. Next, a 4.5‐mm tunnel is drilled over the pin. Tunnel length is measured (Figure 1) and marked as appropriate on the graft/loop sutures (Figure 2). Next, a tunnel of approximately 20 mm is made, reflecting the measured diameter of the prepared graft. The tunnel is then debrided and cleared of remaining bone debris and evaluated (Figure 3a). Passing sutures are passed using the beath pin. Next, using a commercial tibial guide, a guidewire is inserted through the anteromedial tibial incision aiming to the anatomic ACL insertion site, using the anterior horn of the lateral meniscus as a reference. An appropriate diameter tunnel is established in the tibia. The aforementioned sutures are then passed through the tibial tunnel, and the graft is introduced to its appropriate location, being held with the selected button on the LFC. The graft is transferred under vision through the arthroscopic portals, tracking the button movement through the femoral tunnel, as demonstrated later in Figure 3b–e. This method allows fixing the button as close as possible to the LFC. No Fluoroscopy is needed during this approach. After the chosen button is believed to have flipped, the graft is pulled from its distal side to confirm that the button has landed on the cortex. Tibial fixation is performed using an interference screw with the knee in full extension, and the remaining graft is additionally fixed with an onlay Staple, in all cases.

### Button measurements

**Figure 4:**
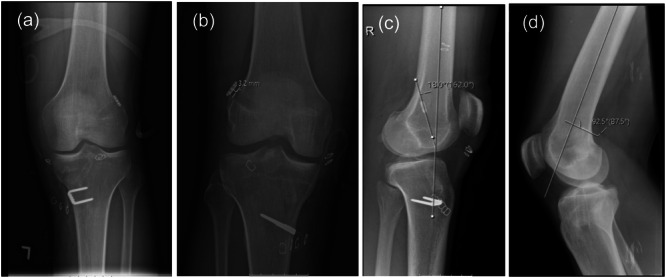
(a–d) Radiographic measurements. (a) An antero‐posterior (AP) X‐ray of unmeasurable distance to the lateral femoral condyle (LFC) using a fixed‐cortical suspension device, calculated as 0 mm. (b) An AP x‐ray demonstrating 3.2 mm button‐LFC distance using fixed‐cortical suspension device. (c) Lateral X‐ray showing the angle between the button orientation and the femoral anatomical line, calculated as 18.0°. (d) Lateral X‐ray demonstrating the button location in the sagittal plane of the femur, which is divided into three equal sections (anterior, middle and posterior). The button is located in the posterior third in this patient.

To assess soft tissue interposition and related parameters, measurements were taken from postoperative X‐rays. The distance between the button and the LFC was measured in millimetres, following the method established by Mae et al. [7] (Figure 4). Distances were further categorised into two groups (≤1 mm and >1 mm according to the aforementioned study). Additionally, the button (sharp) angle relative to the femoral shaft, and the button's relative position on the femur (anterior/middle/posterior), as described by Ozbek et al. [10], were evaluated using the most recent X‐rays. Three orthopaedic surgery residents (A.G., I.M. and Y.K.) performed all measurements after specialised training and under the guidance of a musculoskeletal radiologist (D.A.), utilising the PACS system (PostDICOM B.V. Boven de Wolfskuil 3A 24, 6049LX Herten the Netherland).

### Statistical analysis

We performed an a priori power analysis to decide on the required sample size for this study. We planned to compare two independent cohorts (fixed‐loop and adjustable‐loop) for difference in a main outcome measurement which is a continuous variable (button to LFC distance). We assumed the mean button to LFC distance in one group would be 1 mm and 2 mm in the other group (with a 1 mm standard deviation). Aiming for 80% power and a significance level (alpha) of 0.05, we calculated we would require at least 32 patients in each group.

We decided to aim for 100 patients in each group to improve our confidence in the accuracy of the results even further.

Demographic characteristics were described based on their distribution. We then compared baseline demographic and study outcome measurements between the two groups. The comparative analysis was performed based on variable types. Categorical variables were compared using Chi‐square tests, while continuous variables were compared using T‐tests. We also performed a post hoc logistic regression to study correlations between outcome measurements with significant differences between the two groups. Inter‐observer correlation was assessed with interclass correlation. The resultant odds ratios (OR) were used as measurements of effect sizes. A *p*‐value of <0.05 was used for statistical significance. Survival analysis has been conducted using Mantel–Cox test, to evaluate survival differences. All statistical analyses were performed using Microsoft Excel 2019 and IBM® SPSS® Statistics for Windows, Version 19.0.

## RESULTS

Overall, 1750 cases were identified, of which 1,506 were excluded for the following reasons: ACL reconstruction with femoral fixation other than a suspensory extracortical button, revision ACL reconstruction, missing or partial operative report, poorly performed X‐rays, multiple ligament reconstructions. Of the remaining 245 cases, 59% of patients underwent femoral graft fixation with a fixed‐loop device, and 41% of patients underwent femoral graft fixation with an adjustable‐loop.

Patient demographics are described in Table 1. Included patients had a mean age of 32 ± 10.6, and 206 (84%) patients were male. The Fixed‐loop group was significantly older, with a mean age of 35.2 compared to 27.5 years (*p* < 0.001). The right knee was involved in 54% of cases and the mean time to surgical intervention from time of injury was 22 ± 46.8 months. Time from injury to surgery was significantly longer in the fixed‐loop button with 27.1 months compared to 14.8 months in the adjustable‐loop button group (*p* < 0.043). The ACL rupture was complete in 90% of cases. Reconstruction with a hamstrings autograft was performed in 91% of cases, and in 9% of cases an allograft was used (most commonly tibialis posterior or anterior). Other concomitant procedures performed are described in Table 1.

**Table 1:** Patient demographics.

	Fixed loop (*n* = 144)	Adjustable loop (*n* = 101)	*p* value
Age (years), M (SD)	35.25 (9.24)	27.48 (10.89)	<0.001
Gender (male), *N* (%)	124 (86%)	82 (81%)	0.300
Knee (right side), *N* (%)	73 (51%)	58 (57%)	0.433
Activity level, *N* (%)			0.102
0	13 (15%)	8 (12%)	
1	62 (74%)	44 (65%)	
2	9 (11%)	16 (24%)	
Time to surgery (months), M (SD)	27.09 (56.59)	14.77 (27.14)	0.043
Graft type, *N* (%)			0.100
Autograft	127 (89%)	93 (95%)	
Allograft	16 (11%)	5 (5%)	
Rupture type, *N* (%)			0.022
Complete	122 (86%)	95 (95%)	
Partial	20 (14%)	5 (5%)	
Meniscectomy, *N* (%)			
Medial	58 (40%)	21 (21%)	0.001
Lateral	48 (33%)	30 (30%)	0.548
Meniscus suture, *N* (%)			
Medial	35 (24%)	29 (29%)	0.440
Lateral	16 (11%)	16 (16%)	0.279
Chondroplasty, *N* (%)	6 (4%)	1 (1%)	0.142
Notchplasty, *N* (%)	70 (54%)	33 (33%)	0.002
Time of follow‐up mo. (SD)	28.27 (36.95)	8.65 (7.79)	0.011

*Note*: Activity level: 0—Not active in sports, 1—recreational athlete, 2—professional athlete.

Abbreviations: %, valid percentage; M, mean; *N*, number; SD, standard deviation.

Patients who underwent fixation with a fixed‐loop device underwent more medial meniscectomies (40% compared to 21%, *p* = 0.001), microfractures (6% compared to 1%, *p* = 0.041), and notchplasties (54% compared to 34%, *p* = 0.002). As described in Table 2, patients in the adjustable‐loop group had significantly wider tibial tunnels drilled as compared to the fixed‐loop group (9.35 mm ± 0.82 vs. 8.69 mm ± 1.02, respectively, *p* < 0.001), and wider femoral tunnels drilled compared to the fixed‐loop group (8.99 mm ± 0.8 vs. 8.32 mm ± 0.94, respectively, *p* < 0.001). A significantly shorter button distance from the LFC was noted in the post‐operative AP X‐ray of the adjustable‐loop button, 0.44 mm ± 0.52 vs. 0.72 mm ± 0.84, respectively (*p* = 0.002). No difference was statistically shown regarding button angle or location in the posterior, middle or anterior thirds of the femoral shaft in the lateral X‐ray. No association was found regarding button distance and future revision or failure of the reconstruction (*p* = 0.285). There was a significant difference in revisions for, or failures of the reconstructed ligament amongst fixed‐loop and adjustable‐loop buttons (17/143 [12%] vs. 4/98 [4%], respectively, *p* = 0.035). A post hoc regression analysis was performed to explore this association. The odds ratio (OR) for a revision, given a higher button distance was 1.361, *p* = 0.219. This model was statistically insignificant (Nagelkerke *R* ^2^ = 0.01, *p* = 0.285).

**Table 2:** Late follow‐up radiographic outcomes.

	Fixed loop *N* = 28	Adjustable loop *N* = 14	*p* value
Tibial tunnel size (mm), M (SD)			
Intra‐operative	8.69 (1.03)	9.35 (0.82)	<0.001
Post‐operative	10.68 (2.31)	12.40 (2.25)	0.024
Delta from last X‐ray	1.55 (2.60)	2.72 (1.89)	0.141
Femoral tunnel size (mm), M (SD)			
Intra‐operative	8.32 (0.94)	8.99 (0.80)	<0.001
Post‐operative	9.72 (1.96)	10.43 (2.02)	0.253
Delta from last X‐ray	1.32 (1.61)	1.19 (1.74)	0.809
LFC‐button distance (mm), M (SD)			
Immediate post‐operative	0.72 (0.84)	0.44 (0.52)	0.002
Late post‐operative	0.33 (0.64)	0.37 (0.59)	0.866
Delta	−0.23 (0.78)	‐0.03 (0.406)	0.363
Button angle (degree), M (SD)			
Immediate post‐operative	43.84 (24.44)	44.58 (25.74)	0.866
Late post‐operative	41.77 (31.79)	45.13 (25.68)	0.772

Abbreviations: LFC, the lateral femoral condyle; SD, standard deviation.

Chi‐square test was made, and no statistical difference was found between autograft Hamstring and Allograft (9.5% and 8.8%, respectively) in regard to failure or revision rates (*p* = 0.911). Another calculation was made to examine failure of Allograft alone with the two femoral fixation devices. No statistically significant difference was found (failure of fixed‐loop with allograft was 11.2% and adjustable‐loop 5.1%, *p* = 0.100).

Statistical analysis was also made in order to compare the change in tibial and femoral tunnel size in follow‐up X‐rays. Of importance, the sample size of patients with follow‐up X‐rays was relatively small (fixed‐loop group *N* = 28, adjustable‐loop group *N* = 14). No statistical difference was found in tunnel width or button migration (as reflected by button‐LFC distance or button‐to‐shaft angle) between the two groups on follow‐up X‐rays (Table 2).

A multilinear regression analysis was conducted to assess variables which may be associated with greater button to LFC distance. Complete/partial ACL tear, meniscectomies, microfractures, notchplasties, tibial and femoral tunnel sizes and button type. Of these, only button type was found to be correlated to LFC‐button distance. Fixed‐loop button was found to have an OR = 2.4 for having > 1 mm LFC‐button distance (Table 3).

**Table 3:** Regression analysis—Odds ratio of belonging to Group 2, having over 1 mm distance from the LFC.

	*B*	SE	Wald	*df*	Sig.	Exp(*B*)	95% CI for EXP(*B*)	
							Lower	Upper
ACLR complete/partial (1)	−0.109	0.491	0.049	1	0.825	0.897	0.343	2.348
MMx (1)	0.223	0.350	0.408	1	0.523	1.250	0.630	2.482
MFX (1)	−0.662	0.861	0.592	1	0.442	0.516	0.095	2.785
NOTCHPLASTY (1)	−0.353	0.320	1.215	1	0.270	0.703	0.375	1.316
**Button type (1)**	0.876	0.361	5.896	1	**0.015**	2.402	1.184	4.874
TIBIA TUNNEL SIZE IN‐OP	−0.525	0.298	3.103	1	0.078	0.592	0.330	1.061
FEMORAL TUNNEL SIZE IN‐OP	0.492	0.307	2.565	1	0.109	1.636	0.896	2.989
Constant	−0.299	1.901	0.025	1	0.875	0.742		

Abbreviations: ACLR, anterior cruciate ligament reconstruction; CI, confidence interval; *df*, degrees of freedom; LFC, the lateral femoral condyle; MFX, microfracture; MMx, medial meniscectomy; SE, standard error.

A statistical analysis has been performed, evaluating inter‐observer agreement amongst 161 patients with appropriate follow‐up. Appropriate correlation between observers was found in evaluating button‐cortex distance (ICC = 0.856). Femoral tunnel degree on AP X‐ray and button degree on LAT X‐ray had only mild correlation, poor correlation was found assessing button position on LAT view (Table 4).

**Table 4:** Inter‐observer correlation analysis—Interclass correlation coefficient (ICC) between observers.

	X‐ray view	ICC	*p* value
Femoral tunnel degree	AP	0.479	<0.001
Button to cortex distance	AP	0.856	<0.001
Button position	LAT	0.035	0.43
Button degree	LAT	0.644	<0.001

Abbreviations: AP, anteroposterior; LAT, lateral, ICC, inter‐observer correlation

**Graph 1:**
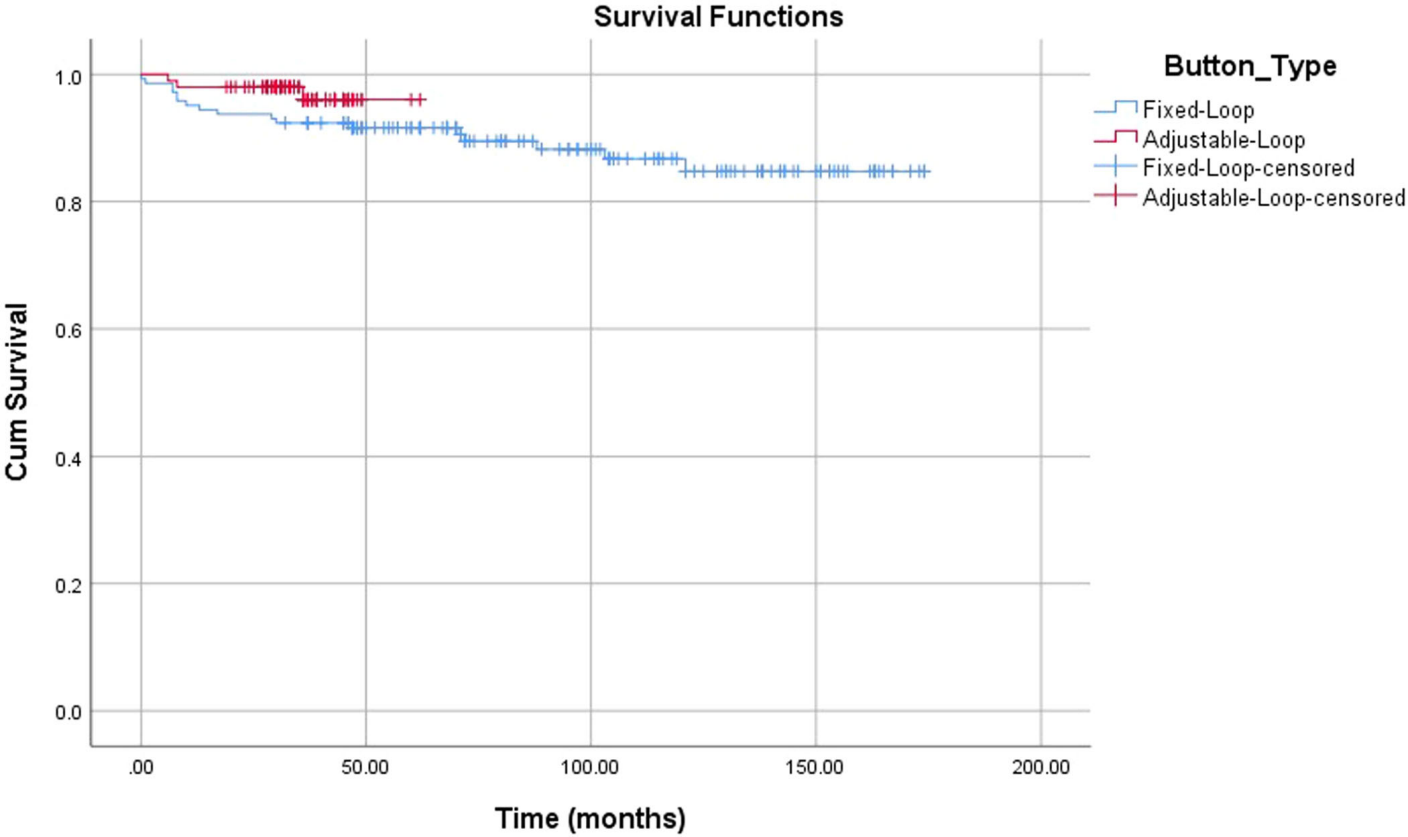
Survival analysis for fixed‐loop and adjustable‐loop cortical suspension devices in the time frame of the study.

Survival analysis has been conducted to study the differences between Fixed‐loop and Adjustable‐Loop cortical suspension device (Graph 1). No significant difference has been found (*p* = 0.149, Table 5).

**Table 5:** Survival analysis.

Overall comparisons			
	Chi‐Square	*df*	Sig.
Log rank (Mantel‐Cox)	2.084	1	0.149
Breslow (generalised Wilcoxon)	2.336	1	0.126
Tarone–Ware	2.221	1	0.136

## DISCUSSION

The main findings of this study are that ACL reconstruction using an adjustable‐loop rather than a fixed‐loop device is associated with shorter LFC‐button distance and lower revision rates. Along with previous literature [10], we did not find a direct association between LFC‐button distance and revision rates.

In a previous meta‐analysis by Shah et al. [11], a comparison was made between different femoral fixation techniques, for ACL reconstruction using hamstrings autograft. Nineteen randomised controlled trials were included. In his study, no statistically significant differences in outcomes were found between the various femoral fixation techniques. This meta‐analysis compared extra‐cortical buttons, interference screws, and trans‐femoral cross‐pins. Another meta‐analysis of 15 cohort studies by Elmholt et al. [3] aimed to compare fixed‐ and adjustable‐loop cortical suspension devices regarding the risk for revision following ACL reconstruction with hamstrings autograft. The authors found no differences in any of the studies included in terms of revision rates, knee stability, or patient‐reported outcomes. However, the quality of evidence was graded “very low.” A large sample population study by Elmholt et al. [4] on 12,723 patients over 15 years old undergoing ACLR using hamstring allograft, proved non‐inferiority to adjustable‐loop in compare to fixed‐loop in regard to revision rates, knee stability assessed via pivot test post‐operatively and patient reported outcome (PROM). In contrast to these studies, we did find a statistically significant difference in revision rates between the two groups, with the adjustable‐loop group showing lower revision rates.

A study by De‐Mees et al. [8] reported a correlation between femoral tunnel position and failure rates, suggesting that drilling the tunnel in the posterior 35% of the Blumensaat line is associated with the best results regarding failure rates. Ozbek et al. [10], however, found no correlation between button location on the lateral view, as a sign of tunnel location, and button migration. In contrast to the study by De‐Mees, and similar to the study by Ozbek, this study has shown no statistically significant correlation between button position on lateral view and failure rates. In regards to the clinical implication of button distance from the lateral cortex, Ozbek et al. [10] have already proposed no clinical implications, which our study had also confirmed, finding no association between soft‐tissue interposition and failure rates, although only a small number of failures and revisions were found in our cohort over‐all. Worth mentioning is that our method for evaluation of button position was based on the method suggested by Ozbek et al. [10] in his study, while the study by De‐Mees et al. [8], compared tunnel position in relation to the Blumensaat line.

A study by Mae et al. [7] tried to assess button migration and its effect on clinical outcomes of a specific Fixed‐loop device (Endobutton, Smith&Nephew Endoscopy, Andover, MA) with or without soft tissue interposition between the button and the lateral cortex. Fixation with soft tissue interposition found to migrate significantly more frequently. However, no clinical impact was found for soft tissue interposition or migration. Our study found resembling results, with no direct association between LFC‐button distance and revision rates.

The brochure for the adjustable‐loop cortical fixation device technique promises better graft protection, fixation strength, and less displacement, compared to fixed‐loop cortical suspension devices. To our knowledge, this is the first clinical study to compare this primary fixed‐loop fixation method and the new‐generation, adjustable‐loop fixation devices in regard to button‐to‐LFC‐button distance and failure rates. Indeed, our results show a lower rate of revision surgeries associated with the newer adjustable‐loop device. However, despite the lower button‐to‐cortex distances we have observed in this device, we were unable to demonstrate a statistically significant association between button distance and revision rates, as shown in previous studies. Worth mentioning the satisfying inter‐observer correlation for button to cortex distance in our study. The lower distance between the button and cortex is theorised to be attributed to the novel technique of this button, its flip technique, making it descend straight on the cortex while flipping, minimising the risk of soft tissue interposition.

It is also important to note that we observed larger tibial and femoral tunnels associated with using adjustable‐loop devices. We believe this resulted from evolving graft preparation techniques in our institution, moving from graft dual folding in the previous years to triple folding in the later years, resulting in thicker grafts. This could have also reduced graft failure rates in this study, which could be an alternative explanation to our results, but as previously mentioned, no statistically significant correlation was found between button to LFC distance and tunnel size (*p* = 0.078 for tibial tunnel and *p* = 0.109 for femoral tunnel, Table 3).

## LIMITATIONS

This study has several limitations. First, we were unable to control the variation in surgeons operating during the study period. Additionally, the surgeons at this single institution had more experience using fixed‐loop cortical suspension devices compared to adjustable‐loop devices. Another limitation is that the adjustable‐loop device was primarily used in the later years of the study, which might suggest that advancements in surgical techniques could have influenced the lower rates of revision and failure. For example, the use of a triple‐folded 6‐strand hamstring graft instead of a double‐folded 4‐strand one, resulting in a wider graft, may have contributed to these improvements. Furthermore, the novel technique of monitoring the button's ascent up the femoral tunnel, as demonstrated in Figure 3, was developed to reduce the likelihood of soft tissue interposition, potentially explaining the lower failure rates in surgeries performed in more recent years. Another limitation is there was no standardisation to the magnification factor of X‐rays. A significant limitation related to this issue is that the later reconstructions may not have reached failure yet, as follow‐up was shorter in the adjustable‐loop group, this limitation can also be seen in the survival analysis at Graph 1, where the fixed‐loop reaches plateau after 120 months of follow‐up while adjustable‐loop is still at active decline A longer follow‐up may reveal a better understanding of the survival of reconstruction. On the other hand, the adjustable‐loop group was younger and likely more active, possibly increasing their risk of failure. Additionally, the time from injury to surgery was shorter in this group. As mentioned earlier, this is a retrospective study. Patient follow‐up was taken by searching medical records for the aforementioned details for statistical analysis. Maybe a prospective study, actively inviting patients to the clinic may reveal more accurate assumptions about failure rates or revision surgeries conducted outside of our institution. Finally, all measurements were conducted on 2D X‐rays, with no standardised size reference, which could have influenced our measurements.

## CONCLUSIONS

In conclusion, as opposed to our previously mentioned hypothesis, the use of adjustable‐loop cortical suspension devices was found to be associated with shorter button‐to‐LFC distances and lower failure rates compared to fixed‐loop devices. However, a direct correlation between shorter button‐to‐LFC distance and failure rate was not found to be significant. Collectively, these results may have a genuine clinical impact on the choice of button fixation, though further studies are necessary to thoroughly investigate these findings.

## AUTHOR CONTRIBUTIONS

**Ron Gilat**: Writing of the article. **Amit Gilad**: Data analyzation; writing of the article; submitting manuscript. **Ilan Y. Mitchnik**, **Yoav Comay**, and **Assaf Moriah**: Data analyzation. **Gabriel Agar** and **Yiftah Beer**: Contributor on writing of the article. **Dror Lindner**: Research advocates.

## CONFLICT OF INTEREST STATEMENT

The authors declare no conflicts of interest.

## ETHICS STATEMENT

An institutional review board (ASF‐0021‐23) approved the study prior to its commencement.

## Data Availability

The data that support the findings of this study are available from the corresponding author, Amit Gilad, upon reasonable request.
